# Reduced Handgrip Strength in Congenital Heart Disease With Regard to the Shunt Procedure in Infancy

**DOI:** 10.3389/fped.2018.00247

**Published:** 2018-09-06

**Authors:** Jan Müller, Leopold Röttgers, Rhoia C. Neidenbach, Renate Oberhoffer, Peter Ewert, Alfred Hager

**Affiliations:** ^1^Department of Sport and Health Sciences, Institute of Preventive Pediatrics, Technische Universität München, Munich, Germany; ^2^Department of Pediatric Cardiology and Congenital Heart Disease, Deutsches Herzzentrum München, Technische Universität München, Munich, Germany

**Keywords:** handgrip, shunting, tetralogy of fallot, congenital heart disease, blalock-taussig-shunt

## Abstract

**Objective:** In many patients with congenital heart disease (CHD) arterial blood flow to the arms is inhibited due to shunt surgery in infancy. This study investigates the handgrip strength of patients with CHD in regard to previous shunt procedures.

**Patients and Methods:** Handgrip was evaluated in 424 patients with various CHD (189 female, age 28.1 ± 13.4 years) including 63 with shunt procedures in infancy; and 123 controls (51 female, 35.6 ± 14.2 years) using a Jamar dynamometer adjusted for hand size. The best of three repetitions was recorded for each side and the right-to-left hand ratio was calculated. The 63 shunted patients were grouped considering the side of the shunt: 14 right, 35 central and 14 left.

**Results:** Patients with CHD, especially shunts, had significantly lower handgrip strength in the dominant hand than controls (controls: 43.2 ± 14.8 kg, CHD: 36.8 ± 14.8 kg, left shunt: 33.6 ± 14.6 kg, central shunt: 30.7 ± 15.2 kg and right shunt 27.8 ± 13.6 kg; *p* < 0.001). In controls the right hand was 8.3% stronger, comparable to patients with either no shunt or central shunt (controls: 8.3 ± 13.2%; no shunt: 7.9 ± 15.3%; central shunt: 9.5 ± 18.1% *p* = 0.820). In patients with a left shunt the right hand was 22.5 ± 17.8% stronger than the left (*p* = 0.027 compared to central) while in those with a right shunt the right hand was 2.3 ± 18.3% weaker (*p* = 0.049 compared to central).

**Conclusions:** Shunt procedures in infancy cause reduced handgrip strength in adulthood and diminished handgrip strength of the ipsilateral site.

## Introduction

Pulsatile arterial blood flow is necessary for organ and muscle development. In many patients with congenital heart disease (CHD) arterial blood flow to the arms is inhibited due to shunt surgeries in infancy. Especially in older generations, patients with Tetralogy of Fallot (ToF) often received a palliative procedure with a Blalock–Taussig shunt (BTS) (1) prior to corrective surgery. Follow-up studies have shown that handgrip strength and arm length of the ipsilateral site is reduced after BTS (2–4). These studies also emphasize a greater reduction in classic BTS compared to modified BTS (3).

As alternatives to BTS, other procedures such as aorto-pulmonary shunts and right ventricle to pulmonary artery (Sano) shunt (5, 6) are the primary step in treating univentricular hearts or Tetralogy of Fallot; subclavian flap aortoplasty is used in patients with coarctation of the aorta (7). All of these procedure cause reduced arterial blood flow which, depending on the type of shunt and the shunt site, affect either the right or left arm, or the central part of the body.

This study investigates the handgrip strength and right-to-left hand ratio of patients with CHD in regard to shunt procedure in infancy.

## Patients and methods

### Study subjects

Grip strength was measured in 421 consecutive patients with various CHD (189 female, 28.1 ± 13.4 years) and 123 controls (51 female, 35.6 ± 14.2 years). The study characteristics are displayed in detail in Table 1. Of those a total of 63 patients (14.9%) underwent a shunt surgery in infancy at a mean age of 2.7 ± 7.1 years. Detailed overview of the shunting procedures is given in Table 2. Severity of CHD was classified according to ACC/AHA 2008 Guidelines (8).

**Table 1:** Patients characteristics.

	**Right Shunt (n = 14)**	**Central Shunt (n = 35)**	**Left shunt (n = 14)**	**No Shunt (n = 361)**	**Controls (n = 123)**	*p*-value^*^
Sex (female / male)	9/5	13/20	9/5	158/203	51/72	0.259
Age (years)	29.4 ± 12.9	24.5 ± 12.4	33.4 ± 13.8	28.1 ± 13.4	30.1 ± 12.1	0.120
Body Mass Index (kg/m^2^)	22.8 ± 5.3	22.9 ± 4.3	22.8 ± 5.0	22.9 ± 4.8	22.7 ± 4.1	0.668
Grip Strength (kg)	27.8 ± 13.6	30.7 ± 15.2	33.6 ± 14.6	35.6 ± 14.6	43.2 ± 14.7	<**0.001**
Right-to-Left Hand Ratio (ratio)	0.98 ± 0.18	1.09 ± 0.18	1.23 ± 0.18	1.08 ± 0.14	1.08 ± 0.15	**0.001**

* *p-value for trend from analysis of variance and a chi-square test for sex only. Significant values are shown in bold*.

**Table 2:** Overview of the shunt types.

**Type of Shunt**	**Number**	**Location**	**Cumulative Prevalence**
Sano Shunt (Right ventricle to Pulmonary Art.)	6 (9.5%)	central	35 (55.6%)
Central aorto-pulmonary shunt	20 (31.7%)		
Waterston or Waterson-Cooley anastomosis	9 (13.3%)		
Classic blalock-taussig shunt	12 (19.0%)	left	14 (22.2%)
Modified blalock-taussig shunt	1 (1.6%)		
Truncus brachiocephalicus with a right aortic arch	1 (1.6%)		
Classic blalock-taussig shunt	5 (7.9%)	right	14 (22.2%)
Modified blalock-taussig shunt	4 (6.3%)		
Modified blalock-taussig shunt from the Truncus brachiocephalicus	4 (6.3%)		
Miscellaneous shunt from the right Art. subclavia	1 (1.6%)		

### Handgrip strength measurement

Handgrip strength was assessed with a Jamar® Hydraulic Hand Dynamometer (Sammons Preston, Bolingbrook, USA, IL). Measurements were performed in sitting position with the shoulder adducted and neutrally rotated, the elbow flexed at 90° and the forearm and wrist in neutral position (9). For both hands, the best of three repetitions was recorded and from these values, the right-to-left hand ratio was calculated.

### Data analyses

Descriptive data was expressed as mean values and standard deviation (mean ± *SD*).

The general effect of a shunt on handgrip strength was assessed by linear regression with strength as outcome and with age, sex, and severity class according to the ACC (8) as covariates. Groups were compared using a one-way ANOVA or chi-square test where appropriate. Significant differences between the three groups (right, central, left) were further tested with an unpaired *t*-test.

All analyses were performed using SPSS 23.0 software (IBM Corp., Armonk, NY, USA). Two-sided *p*-values < 0.05 were considered significant.

## Results

As seen in Table 1, handgrip strength was highest in controls with 43.2 ± 14.7 kg followed by patients without shunt (35.6 ± 14.6 kg, *p* < 0.001 compared to controls) and patients with shunt (30.7 ± 14.7 kg, *p* = 0.011 compared to patients without shunt). In shunted patients, those with a left shunt had a higher grip strength than those with either central or right shunt (left: 33.6 ± 14.6 kg, central: 30.7 ± 15.2, right: 27.8 ± 13.6 kg; ANOVA: *p* < 0.001).

After correction for age, gender and severity class in a multivariate regression model, presence of a shunt was independently associated with a lower handgrip strength of 4.5 kg (B = −4.5, beta = −0.088, *p* = 0.037) in patients with CHD compared to controls.

### Comparison between right and left hand (right-to-left ratio)

In controls, the right-to-left ratio was 1.083 ± 0.132, which means the right hand was 8.3% stronger than the left. In patients with a left shunt the right hand was significantly stronger compared to controls (1.224 ± 0.178%; *p* < 0.001). In patients with a right sided shunt the right-to-left ratio switched and it was even the left hand stronger compared to controls (0.979 ± 0.183; *p* = 0.008). Nevertheless, there were no significant differences when comparing controls to patients without shunt (1.079 ± 0.153; *p* = 0.781) or central shunt (1.095 ± 0.181; *p* = 0.672).

**Figure 1:**
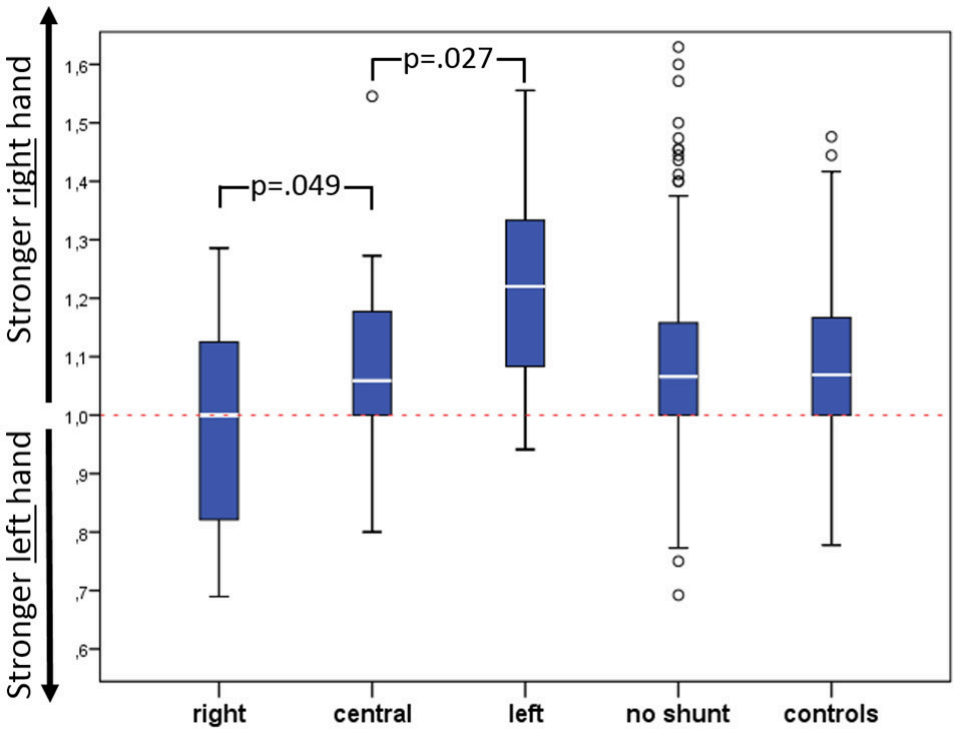
Right-to-left hand ratio of grip strength according to shunt side including p-values for comparison of right/central and left shunt.

Figure 1 specifically illustrates the differences in patients with a shunt. In left shunts, the right-to-left ratio was highest with 1.225 ± 0.178, in central shunt 1.095 ± 0.181 and in right shunts 0.979 ± 0.183. Hence it can be noted that the right-to-left ratio declines progressively when the location of the shunt moves from left to central to right.

## Discussion

Handgrip strength in patients with CHD is diminished, especially in patients that received a shunt in infancy. Moreover, the handgrip strength of the ipsilateral site of the shunt is reduced.

In accordance to other reports (2, 4, 9) we found a reduced handgrip strength of patients with CHD compared to a reference cohort. This muscular deconditioning seems to be multifactorial and it is suggested that reduced physical activity (9, 10), with inappropriate exercise prescription and overprotection (11, 12) or even a generalized myopathy in patients with CHD are contributing factors (13). Other reports (3, 4) in ToF point to another, more simple, explanation for diminished handgrip strength in patients with CHD by associating the handgrip strength to the presence of a shunt surgery in infancy causing limited blood supply to the arm (14). However, our study outlines already reduced handgrip strength in patients with CHD even if they had not received any kind of shunt procedure.

Nevertheless, the handgrip strength of the dominant hand declined progressively from patients with a left sided shunt, to those with a central shunt, to those with a right shunt. Since in general the right hand is the dominant one (4, 15) [as seen in our controls and in patients without shunt (Figure 1)] in patients with a left shunt the handgrip strength of the right hand is not significantly altered because the pulsatile flow is reduced only in the left arm: this increased the right-to-left ratio but did not affect the strength of the dominant (right) hand. Contrarily, patients with a central shunt have a similar right-to-left ratio, similar to controls and CHD patients without shunt: however the central shunt seems to affect the total handgrip strength, which was lower than either controls or patients without shunt. We could only speculate that central shunt lower the pulsatile flow to both arms, leading to a slight reduction in grip strength. Finally, patients receiving a right shunt show the lowest handgrip strength. The missing blood flow to the right arm equalized the strength of the right arm to the left one. Provocatively speaking, right sided shunts make right-handers into left-handers, at least in regard to handgrip strength.

Le Gloan et al. (3) suggest that a modified BTS may ease the severity of the difference in right-to-left ratio. Likewise, in our group the 17 people with a classic BTS (12 left, 5 right) showed the highest deviation in right-to-left hand ratio compared to either controls or patients without shunt, whereas modified BTS, no matter if right or left, resulted in nearly normal right-to-left ratios.

## Conclusion

Handgrip strength of patients with CHD is more reduced in patients with a shunt compared to health controls and is also associated with a weakening of the ipsilateral hand. Central shunts or modified BTS that preserve pulsatile flow appeared to provide a better outcome in regard to strength development and right-to-left hand ratio.

## Study limitations

Grip strength may be a reflection of overall well-being and the absence of data regarding limb dexterity limits the conclusions that can be drawn regarding shunt side. Overall functionality of the limb, such as range of motion and limb length discrepancy were missing, which could had effected overall strength. Albeit significant, the sample size for the right and left sided shunt group is rather low, making a detailed analysis on differences between modified BTS and classic BTS impossible.

## Compliance with ethical standards

The study was approved by the local ethical board of the technical university of Munich and in accordance with the declaration of Helsinki (revision 2008). Patients agreed to the anonymous publication of their data by written informed consent.

## Author contributions

JM and AH were responsible for conception and design of the study. They sampled the data in the study center and were responsible for data monitoring, integrity and analysis, and drafted the manuscript. RN and LR also sampled parts of the data and provided all the necessary information regarding shunting from the patient files. PE and RO gave important input for revising the manuscript. All authors have read and approved the final version of the manuscript.

### Conflict of interest statement

The authors declare that the research was conducted in the absence of any commercial or financial relationships that could be construed as a potential conflict of interest.
